# Analysis of xylem sap proteins from Brassica napus

**DOI:** 10.1186/1471-2229-5-11

**Published:** 2005-06-21

**Authors:** Julia Kehr, Anja Buhtz, Patrick Giavalisco

**Affiliations:** 1 Department Lothar Willmitzer, Max-Planck-Institute of Molecular Plant Physiology, 14424 Potsdam, Germany

## Abstract

**Background:**

Substance transport in higher land plants is mediated by vascular bundles, consisting of phloem and xylem strands that interconnect all plant organs.

While the phloem mainly allocates photoassimilates, the role of the xylem is the transport of water and inorganic nutrients from roots to all aerial plant parts. Only recently it was noticed that in addition to mineral salts, xylem sap contains organic nutrients and even proteins. Although these proteins might have important impact on the performance of above-ground organs, only a few of them have been identified so far and their physiological functions are still unclear.

**Results:**

We used root-pressure xylem exudate, collected from cut *Brassica napus *stems, to extract total proteins. These protein preparations were then separated by high-resolution two-dimensional gel electrophoresis (2-DE). After individual tryptic digests of the most abundant coomassie-stained protein spots, partial peptide sequence information was deduced from tandem mass spectrometric (MS/MS) fragmentation spectra and subsequently used for protein identifications by database searches. This approach resulted in the identification of 69 proteins. These identifications include different proteins potentially involved in defence-related reactions and cell wall metabolism.

**Conclusion:**

This study provides a comprehensive overview of the most abundant proteins present in xylem sap of *Brassica napus*. A number of 69 proteins could be identified from which many previously were not known to be localized to this compartment in any other plant species. Since *Brassica napus*, a close relative of the fully sequenced model plant *Arabidopsis thaliana*, was used as the experimental system, our results provide a large number of candidate proteins for directed molecular and biochemical analyses of the physiological functions of the xylem under different environmental and developmental conditions. This approach will allow exploiting many of the already established functional genomic resources, like i.e. the large mutant collections, that are available for Arabidopsis.

## Background

The higher plant body consists of functionally specialized organs such as leaves, stem, fruits, flowers, and roots. Because plants are immobile and have to cope with changes in their environment, interaction of different organs is essential to coordinate growth, development and defence reactions also between the most distant plant parts [1]. Transport of nutrients and information molecules over long distances is, in most instances, mediated by the vascular bundles that mainly consist of xylem and phloem. The xylem constitutes a channel system for water and inorganic nutrient transport from roots to above-ground plant organs. Xylem transport occurs through the dead and hollow xylem vessels that belong to the apoplastic space. In addition to inorganic salts, organic nutrients, such as amino acids, sugars, and organic acids are translocated through the xylem from roots to aerial organs [2-4]. The above-ground plant parts are dependent on the inorganic and organic compounds that are taken up or produced by the roots and distributed by the xylem network. A specific example of root-produced organic compounds that are translocated in xylem sap are plant hormones (i.e. cytokinin, abscisic acid, auxins, gibberellins), which are known to be important in the control of different aspects of plant development in above-ground organs [1]. For example, they are involved in the coordination between root and shoot differentiation, growth, and development [5-9].

Earlier reports have already described the presence of proteins in the xylem sap of numerous plants, like watermelon [10], apple, peach, pear [11], cucumber [12], squash [13], rice [14], and tomato [15] and recently, biochemical approaches have revealed the identities of a few of these xylem sap proteins. Peroxidases and chitinases [11,16,17], pathogenesis-related (PR) proteins [15], a glycine-rich protein [18], a cysteine-rich protein [19] and a 30 kD lectin [12] have been found. It is speculated that some of these proteins might exert specific physiological functions in aerial organs [13], although the biological significance and the regulation of these proteins are not fully understood [1]. It has been shown that xylem protein patterns change in response to infection by pathogenic fungi [15,19] and there are indications that interactions between proteins and pathogens within the xylem vessels, at least partly, determine the grade of resistance or susceptibility of tomato plants towards the vascular wilt pathogen *Fusarium oxysporum *[15]. Also after bacterial infection in rice, a xylem peroxidase was described to accumulate in xylem vessels [14]. However, further detailed evidence supporting the role of xylem sap proteins in plant defence reactions is so far missing.

Recent results indicate that the expression of xylem proteins can be highly regulated also by other factors than pathogen invasion. The root-specific expression of 30 kD xylem sap protein (XSP30), for example, is controlled by a circadian clock and shows diurnal fluctuations. This protein appears to be influenced additionally by unknown gibberellin-induced mediators that are produced by leaves and transported to roots to influence XSP30 expression [1].

Another important issue is the origin of xylem sap proteins, because xylem vessels are dead cells that are incapable of transcription and translation. Proteins may reach the xylem sap either specifically or they could originate from developing tracheary elements or flushed away from adjacent parenchyma cells [11] or the vessel cell walls. Currently, there is no data on the synthesis sites of most xylem sap proteins available. The few proteins analyzed so far appear to be expressed root-specifically in xylem parenchyma and pericycle cells and are supposed to be actively secreted into xylem sap by root cells, as has been shown previously for XSP30 [12] and two glycine-rich xylem sap proteins [20]. The secretion of proteins into xylem sap is, like for other apoplastic proteins, most likely mediated by an amino-terminal signal peptide [21,22], which has been detected in the sequences of most of the thus far known xylem sap proteins [12,16,18,19].

Most recent approaches to analyze xylem sap proteins have been performed by low resolution one-dimensional polyacrylamide gel electrophoresis (1-DE) and resulted in the identification of a limited number of xylem specific proteins from different plant species [15,16,19]. Based on this lack of comprehensive protein information of the xylem sap, the aim of the present study was to provide an overview of the proteins present in this plant specific transport fluid, by separating them on high-resolution two-dimensional (2-DE) polyacrylamide gels and subsequently identify a substantial number by tandem mass spectrometry (MS/MS).

## Results and discussion

### Xylem protein extraction, separation and identification

In this study, proteins from xylem sap of adult *Brassica napus *plants were precipitated by acetone, separated by 2-DE and partial amino acid sequences were determined by tandem mass spectrometry. As demonstrated before, the xylem sap collected with the employed method showed no detectable contaminations from phloem sap or other adjacent cellular compartments, if the cut stems are thoroughly rinsed with water before starting the sample collection [16]. In addition, the xylem sap protein patterns of *Brassica napus *plants were clearly distinct from 2-DE spot patterns derived from purified phloem sap or whole *Brassica napus *stem tissue protein extracts (unpublished data).

**Figure 1:**
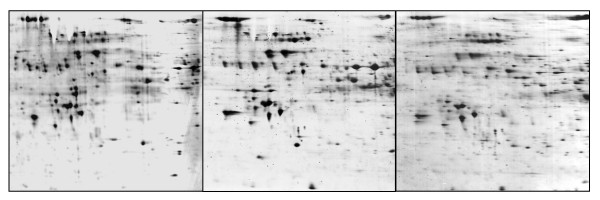
**Reproducibility of xylem sap 2-DE patterns**. 2-DE patterns of xylem sap proteins from three independent sets of flowering, 12-week-old *Brassica napus *plants that were grown independently during a period of eight month. The left hand image shows the protein gel represented in Figure 2.

**Figure 2:**
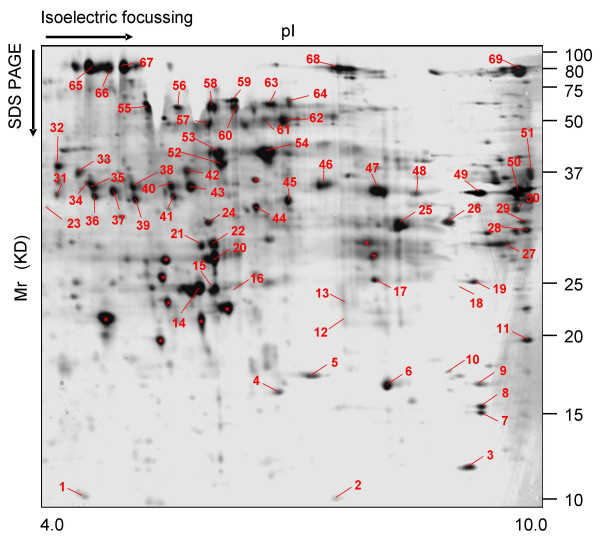
**Protein identifications from a representative xylem sap 2-DE gel**. Representative 2-DE gel of xylem sap proteins from 12-week-old *Brassica napus *plants. Proteins were identified by partial sequences determined by tandem mass spectrometry. Numbers indicate spots from which proteins could be identified. Sequences and identifications are listed in Table 1. Asterisks indicate spots that were analyzed but led to no or low quality mass spectra that did not allow protein identifications. Molecular masses in kD are indicated on the right hand side of the gel.

Coomassie staining of the 2-DE gels from this root pressure exudate allowed visualization of approximately 300 protein spots (Figures 1 &2). In the presented experiments the most intense spots, which could be reproducibly retrieved from several independent xylem sap protein extractions (Figure 1), were excised from 2-DE gels, digested *in situ *with the site-specific protease trypsin, before partial amino acid sequences were determined by tandem mass spectrometry, followed by database searches for protein identification. Using this approach, a number of 69 protein spots could be reliably identified from at least two independent gels by the high similarity of the determined partial amino acid sequences to plant proteins in the NCBI plant protein database (Figure 2, Table 1).

Most of these proteins, 64 protein spots, matched to proteins from the fully sequenced genome of the model plant *Arabidopsis thaliana*. This was not unexpected, since the genomes of *Arabidopsis thaliana *and *Brassica napus *show a very high degree of identity [23]. The other 5 proteins either directly corresponded to database entries from *Brassica napus*, or matched to database entries from *Brassica rapa *or rice (Table 1).

The observed molecular masses of the majority of the identified proteins matched well to the theoretical molecular weights predicted from the amino acid sequences of the database entries (Table 1). Only a few proteins showed significant differences between the theoretical and the observed masses, i.e. the protein spots for the curculin lectins (spots 35, 36, 38–41) showed significantly lower molecular weight on SDS PAGE gels than expected for the Arabidopsis homologues the identification was based on (Table 1). This could indicate on the one hand that some of the *Brassica napus *curculin lectins are smaller than their Arabidopsis homologues or that they, as has been shown for other proteins [24,25], show a higher mobility in SDS PAGE gels than predicted. Alternatively, these Brassica curculin lectins might be products of proteolytic processes trimming these gene products to a smaller size. Additionally it could be observed that most of the proteases identified from the xylem sap showed lower than predicted molecular weights on the 2-DE gels (spots 8, 11, 19, 34). In the case of the identified subtilases, two forms with different observed masses, namely one of about 56 kD (spots 55, 56, 59, 60, 63), which represents a smaller than predicted isoform and one of 80 kD (spots 65–67), which corresponds to the expected mass of the identified protein, were observed. These differences might be explained by the fact that proteases often show auto-proteolytic activity that results in molecules of different sizes, unprocessed large pro-proteases and proteolytically processed smaller proteins. It has been shown for one subtilases that this step is needed to activate the enzyme [26].

In contrast to the observed smaller than expected proteins, one of the identified germin-like proteins (spot 69) displayed a higher molecular mass than expected, which might be due to the reported observation that this class of proteins is known to occur as oligomers *in vivo *[27]. Alternatively, as has been shown for other proteins [28-30], this germin-like protein could show an abnormal, reduced mobility in the SDS PAGE separation.

Analyzing the amino acid sequences of the homologous proteins derived from the plant protein database searches, revealed a common characteristic that is probably essential for the apoplastic localization of these proteins: with the exception of 2 proteins (ubiquitin in spots 1 & 2 and beta-1,3-glucanases in spots 49 & 50), all identified proteins are likely targeted to the secretion pathway when analyzed by SignalP, a program that predicts N-terminal peptides and signal peptidase I cleavage sites [21,22]. This observation is in full agreement with previous results of xylem sap proteins from different plant species, underlining that xylem sap proteins belong to the class of secreted proteins [15,16].

While some of the identified proteins appeared to be present in only one single protein spot (PR1a, spot 4; thaumatin, spot 18; a cyclase family protein, spot 44; polygalacturonase inhibitor, spot 51), in some cases the partial sequences led to the identification of the same protein in two or more different protein spots of similar molecular weight, but with a variable isoelectric point (ubiquitin, spots 1 & 2; beta-glucanases, spots 49 & 50; pectin methylesterase inhibitor, spots 6 & 9; xyloglucan:xyloglycosyl transferase, spots 21 & 22; polygalacturonase, spots 58 & 64). The occurrence of similar or identical gene products in different spots is a common feature observed in 2-DE analysis and can be caused by post-translationally modifications of distinct amino acids of one single gene product [31]. Alternatively, since our MS analysis does not provide full sequence coverage of the identified proteins, these protein spots can represent products from highly similar, but distinct genes of a gene family. The appearance of multiple protein spots, derived from different but functionally or sequence-related protein families, could also be observed for the glycine-rich cell wall proteins (spots 3, 5, 13); the germin-like proteins (spots 17 & 69), the chitinases (spots 14, 15, 16, 20, 23, 27, 29); the lectins (spots 25, 26, 28, 35, 36, 38–41, 57, 61, 62); the peroxidases (spots 24, 30–33, 37, 42, 43, 45, 46–48, 52–54) and for some proteases (spots 7, 8, 11, 19, 34, 55, 56, 59, 60, 63, 65–68), indicating a certain degree of redundant expression for specific gene products.

### Possible functions of the identified xylem sap proteins

#### Peroxidases

One protein family detected in our xylem 2-DE gels comprises peroxidases. This class of proteins has a multitude of possible functions [32], including the generation of reactive oxygen species and the regulation of H_2_O_2 _levels *in planta*. It has been shown that peroxidases can be involved in plant cell wall strengthening by different mechanisms, e.g. by cross-linking and polymerizing proline-rich proteins in the cell wall [33,34] or by catalyzing lignin deposition [35]. Interestingly, this family of proteins have been found in xylem sap from all plants that have been analyzed so far [11,16]. In the current analysis, peroxidases were the largest protein group, containing 6 different proteins, derived from a total of 15 different protein spots (spots 24, 30–33, 37, 42, 43, 45, 46–48 and 52–54). Apart from the previously mentioned cell wall dependent activities, a large number of additional possible functions were attributed to this ubiquitous protein family [34].

#### Proteases

Another large and functionally diverse group of proteins within the plant genome are the proteases. They represent the second largest functional group identified from our 2-DE gels, with 14 identified protein spots (7, 8, 11, 19, 34, 55, 56, 59, 60, 63, 65–68), representing 6 different, unrelated proteases. The eclectic mix of proteases identified from rape xylem sap represents a cross section of the large protease repertoire in plants. For example, coding sequences for a number of more than 550 different potential proteases, which are grouped in more than 50 different families, are found in the Arabidopsis genome [36]. The proteases identified from our analysis could be grouped into 5 different protease families: S8 (subtilisin-like serine proteases, spots 59, 60, 63, 65–67, 68), S10 (serine carboxypeptidase, spot 34), C1A (papain-like cysteine proteases, spots 7, 8), A1 (pepsin-like aspartic acid proteases, spot 11) and T3 (threonine proteases, spot 19). Only a few of the proteases identified in the current study have been previously characterized from other plant species. It was shown that a homologue of the cucumisin-like S8 subtilisin protease ARA12 (spot 68) is involved in actinorhizal nodule development in *Alnus glutinosa *(European alder) roots [37], while the Arabidopsis homologue seems to be expressed more ubiquitously, with a certain specificity in silique development [37]. The reports concerning other subtilisin-like proteases demonstrate their importance in the regulation of developmental processes, like the distribution and density of stomata on *Arabidopsis thaliana *leaves [26,38]. A function in xylem development could be associated with the C1A papain-like protease, XCP2, which was identified from 2 protein spots from our 2-DE gels (spots 7, 8). This protein and its close homologue XCP1 were shown to be expressed in xylem tissue of Arabidopsis [39] and these proteins are thought to be involved in xylem formation.

For the other proteases identified from the xylem sample nothing, except from the facts that they all contain specific protease domains and that they contain a secretion pathway sequence, explaining their presence in the xylem, is known.

#### Defence-related proteins

One group of proteins that has been closely associated with plant defence are the pathogenesis-related (PR) proteins [35]. In our 2-DE gels we detected a single, low MW protein spot (spot 4, Figure 2) that is similar to a protein belonging to the family of PR1 proteins. This result confirms the previously observed occurrence of a PR-1a-like protein in fungus-infected tomato xylem sap [15], although in our study the rape plants were not actively challenged with pathogens. The PR1 family proteins were the first identified PR proteins and show antimicrobial properties [40]. However, the molecular or biochemical basis by which these proteins provide this function remained up to date elusive.

Another class of proteins that is often associated to the PR proteins are beta-1, 3-glucanases (BGs) and chitinases, which are believed to mediate defence responses because of their potential to degrade fungal cell walls [41]. In our analysis we found 3 different putative endochitinases, belonging to the two different classes I and IV, in a total of 7 different protein spots (spots 14, 15, 16, 20, 23, 27 and 29 in Figure 2), while one BG protein could be identified within two different protein spots (spots 49 & 50), indicating a possible co-transcriptional or post-translational modification of these proteins. The occurrence of chitinases and chitinase activity in xylem sap of different plant species has been observed in earlier studies [16,17], while BG proteins were found thus far only in tomato [11,15].

Interestingly, chitinases and glucanases have been suggested to act in a synergistic manner with thaumatin-like proteins (spot 18) that can bind to β-1,3-glucans [42] and have not been described to occur in xylem sap of healthy, unchallenged plants before, while they were found after fungal infection [15].

#### Lectins

Lectins are carbohydrate-binding proteins that can bind glycans of glycoproteins, glycolipids, or polysaccharides with high affinity. It is assumed that lectins play fundamental biological roles in plants because they are found in many different species and in many different organs and tissues [43].

Legume lectins (spots 25, 26, 28) are one of the largest lectin families with more than 70 lectins reported [44]. Functionally these proteins specifically recognize diverse sugar structures and mediate a variety of biological processes such as cell-cell and host-pathogen interactions and innate immune responses [45]. Curculin lectins (spot 38–41, 57, 61, 62) are, similar to TLPs, sweet-tasting proteins, which often maintain possible mannose-binding sites. Nevertheless, earlier studies have shown that the three mannose-binding sites of curculin from *Curculigo latifolia *are devoid of mannose-binding activity [46] indicating that curculin lectins might have different additional thus far unknown functions.

### Cell wall metabolism and remodelling

The other 20 protein spots identified from Brassica xylem sap have predicted functions probably connected to cell wall metabolism and remodelling.

The proteins found in our study represent a set of proteins discussed to be involved in cell wall stabilization and repair, like the glycine-rich proteins (spots 3, 5, 10) [20,47,48], and the multi-functional xyloglucan:xyloglycosyl transferases (spot 21 & 22), which have all been shown to modify cell wall structure during growth or stress responses [49].

In addition, polygalacturonases (spots 58 & 64) and polygalacturonase-as well as pectin methylesterase inhibitors (PGIPs, spot 51 & PMEIs, spots 6 & 9) have been detected in the xylem sap samples analyzed in this study. While there is not much known about a possible roles of PMEIs in plants, the widespread PGIPs have been thoroughly investigated in different plant species [50]. PGIPs are typically induced by pathogen infection and stress-related signals [50,51]. Usually they are effective only against fungal PGs and do not influence endogenous plant PGs [52].

## Conclusion

The present study demonstrates that *Brassica napus*, due to its high gene sequence identity to the model plant *Arabidopsis thaliana *[23], provides an excellent source for the large-scale analysis of xylem sap proteins. In the course of our analysis, 69 abundant xylem sap proteins were successfully identified. Nearly all of these proteins contained a N-terminal sequence, targeting them to the secretion pathway [53], which correlates to the fact that the xylem is a part of the apoplastic space [5].

The mixture of rape xylem sap proteins identified in the present study is composed of proteins with various potential functions. In addition to a large number of peroxidases and proteases, different potentially defence-related proteins, lectins, and a number of proteins involved in cell wall modification, remodelling and strengthening could be detected. Further experimentation, employing biochemical and immuno-histochemical analysis of the identified proteins, in conjunction with enzyme assays, will be needed to dissect the precise physiological functions of these xylem sap proteins. This attempt should be largely facilitated by the close relatedness of *Brassica napus *to Arabidopsis, where a lot of functional genomic resources are already accessible.

## Methods

### Plant material

*Brassica napus *plants (cv. Drakkar, Serasem GIE, la Chapelle d'Armentiers, France) were grown in 19 cm pots containing steam-sterilized soil (Einheitserde^® ^Typ T) in a greenhouse under controlled conditions (16 h light, 8 h dark, 25°C day, 20°C night, 55% relative air humidity). Plants were automatically watered thrice a day with tap water containing Hakaphos^® ^spezial as a fertilizer.

### Sample preparation

Xylem samples were obtained after cutting stems of flowering 12-week-old plants, approximately 5 cm above soil level. After thorough washing of the surface on the root side with distilled water, they were blotted dry with filter paper and the exuding fluid was then collected with a hand held pipette until sufficient sample volumes (usually 9–12 ml) were obtained. Aliquots of 3–4 ml xylem sap were each collected from 5 plants in parallel and immediately expelled into 7 ml of precipitation solution [90% (v/v) acetone, 10% (v/v) methanol, 10 mM DTT] provided in falcon tubes on ice and precipitated over night at -20°C. The collection of sufficient xylem sap usually took about 30 min. The precipitated proteins were collected by centrifugation for 15 min at 4000 g at 4°C, washed with acetone, the supernatant was discarded and the pellet was air-dried.

In parallel, protein concentrations were determined omitting acetone precipitation with the Bradford method (BioRad, Munich, Germany), using 100 μl of xylem samples.

### Gel electrophoresis

For 2-DE, the precipitated xylem sap proteins (~120 μg) were re-suspended in 50 μl first dimension buffer [2 M thiourea, 7 M urea, 4% 3-[(3-chloramidopropyl) dimethylammonio]-1-propanesulfonate (CHAPS) and 10 mM DTT]. The following protein separation was performed in a 2-DE system described previously [54,55] but in 16 cm times 1.5 mm tube gels for the isoelectric focusing and 18 × 16 × 0,1 cm SDS polyacrylamide gels in the second dimension.

After protein separation, the gels were stained with coomassie brilliant blue G250 according to [55]. Molecular weights were calculated from a run of the PrecisionPlus All Blue Protein Standard (BioRad) performed on a separate gel.

### Mass spectrometry, database searches and sequence analysis

Sample pre-treatment, trypsin digests and determination of partial amino acid sequences was performed as described previously by Walz. *et al *[56]. In short, protein spots were excised, destained, dehydrated, and digested overnight with modified Trypsin (Roche Diagnostics, Mannheim, Germany). Proteolytic peptides were extracted by trifluoroacetic acid/acetonitrile. The extracts were then vacuum dried and desalted on C_18 _pipette tips (Millipore) before analysis by tandem MS in an electrospray ionization quadrupole time-of-flight tandem mass spectrometer (Q-TOFMS, Micromass, Altrincham, UK), resulting in peptide fragmentation spectra. Raw fragmentation spectra were recalculated with the MaxEnt3 algorithm and then partial sequences were manually deduced using the PepSeq software from Micromass (Altrincham, UK). Only peptides that appeared in digests of the same spot from at least two different gels were taken into account.

Database similarity searches with the derived sequence stretches were performed using the short sequence blast algorithm  against the non-redundant protein database limited to green plants. If several partial amino acid sequences could be obtained, the combined sequences were used for an additional BLAST search to calculate BLAST E-values (the resulting E-values are listed in Table 1). Results with BLAST E-values < E-5 were regarded as being reliable protein identifications. Since E-values are not necessarily meaningful for short sequences, we additionally regarded proteins as identified, from which only a single partial sequence could be obtained that missed the E-value threshold, but that showed a stretch of at least 12 amino acids identical to the corresponding database sequence.

Cleavage sites for signal peptidase I, characterizing secreted proteins, were predicted with SignalP  version 3.0 [21,22].

## Authors' contributions

JK participated in the design and coordination of the study, in MS data processing, database searches and writing of the manuscript. AB carried out the mass spectrometric measurements. PG optimized and performed high-resolution 2-DE and participated in the design of the study as well as in manuscript preparation.

All authors read and approved the final version of the manuscript.

## Figures and Tables

**Table 1:** List of identified xylem sap proteins from Brassica napus. Identifications of xylem sap proteins from the 2-DE gel shown in Figure 2. Sequences were determined from fragmentation spectra obtained by ESI-Q-TOF tandem MS. The resulting partial amino acid sequences were used for database searches with a BLAST algorithm optimized for short sequences. The isobaric amino acids isoleucine (I) and leucine (L), although not distinguishable by MS, are displayed as specified in the corresponding database sequences. BLAST E-values were determined using all non-redundant peptide sequences from one protein spot for another BLAST search. Bold letters in the partial sequences indicate amino acids identical to the database entry. Results of N-terminal secretion sequence predictions are indicated in the right column (Y= secretion sequence predicted, N= no secretion sequence predicted), * indicates that the observed molecular mass was lower and ** indicates that the observed mass was higher than expected.

**Spot No**	**Similarity to**	**Organism**	**Acc. No.**	**Sequence**	**identity**	**E value**	**expected Mr**	**observed Mr**	**aS**
1	**Ubiquitin**	Arabidopsis thaliana	At2g47110	**TITLEVESSDTIDNVK**	16/16 (100%)	9.00E-08	8525	12064	N
2	**Ubiquitin**	Arabidopsis thaliana	At2g47110	**TITLEVESSDTIDNVK**	16/16 (100%)	9.00E-08	8525	11944	N
3	**Glycine-rich cell wall protein**	Arabidopsis thaliana	At4g30450	**AGSSAGSFAGSR**	12/12 (100%)	0.011	9530	13863	Y
4	**Pathogenesis-related protein PR1a**	Brassica napus	AAB09587	**SPQDYVNAHNQAR**	13/13 (100%)	7.00E-39	17532	18145	Y
				**LAAYAQNYADR**	11/11 (100%)				
				**QDSPQDYVNAHNQAR**	15/15 (100%)				
				**GPVQWDGTLAAYAQNYADR**	19/19 (100%)				
				**AGSSADFSGVSAVNLWVNEK**	20/20 (100%)				
5	**Glycine-rich cell wall protein**	Oryza sativa	BAD62284	**GGGYGEGGGYGGG**	15/16 (94%)	2.00E-10	14152	17822	Y
				**GGGY**W**GGGYGGGYGGG**					
6	**Invertase/pectin methylesterase inhibitor**	Arabidopsis thaliana	At1 g47960	**GSSADTSGLALIL**V**DK**	15/16 (94%)	2.00E-32	22326	18297	Y
				**GVID**A**GVEAAV**	10/11 (91%)				
				**ETPDFNLCV**AC**PLDSDPR**	15/18 (83%)				
				**GSSADTSGLALIL**V**DKIK**	17/18 (94%)				
				**ADVPEAIE**CC**SK**	10/12 (83%)				
				**FGEDGVIDAGVEA**A**V**	14/15 (93%)				
7	**Papain-type cysteine proteinase XCP2**	Arabidopsis thaliana	At4g35350	**LDHGVAAVGYGSSK**	14/14 (100%)	4.00E-18	39618	16,603*	Y
				**ALAHQPVSVAIEASGR**	16/16 (100%)				
				**G**V**DLDHGVAAVGYGSSK**	16/17 (94%)				
8	**Papain-type cysteine proteinase XCP2**	Arabidopsis thaliana	At4g35350	**LDHGVAAVGYGSSK**	14/14 (100%)	4.00E-18	39618	16,977*	Y
				**ALAHQPVSVAIEASGR**	16/16 (100%)				
				**G**V**DLDHGVAAVGYGSSK**	16/17 (94%)				
9	**Invertase/pectin methylesterase inhibitor**	Arabidopsis thaliana	At1g47960	**GSSADTSGLALIL**V**DK**	15/16 (94%)	9.00E-38	22326	18297	Y
				**ETPDFNLCV**AC**PLDSDPR**	15/18 (83%)				
				**GSSADTSGLALIL**V**DKIK**	17/18 (94%)				
				**ALDEC**AS**R**	6/8 (75%)				
				**ADVPEAIE**CC**SK**	10/12 (83%)				
				**FGEDGVIDAGVEA**A**V**	14/15 (93%)				
10	**Glycine-rich protein**	Arabidopsis thaliana	At4g30460	**GEGGGYGGGYGGG**G**D**	14/15 (93%)	1.00E-04	13738	19195	Y
11	**Aspartyl protease family protein**	Arabidopsis thaliana	At5g07030	**LPP**S**AIAFNP**A**TGAGTIFDSGTVYTR**	24/26 (92%)	3.00E-15	46919	21,384*	Y
				**VLIDLPN**S**R**	7/9 (77%)				
12	**Disease resistance response/ dirigent protein**	Arabidopsis thaliana	At1g64160	**VIFDDPVTLD**K	8/12 (67%)	3.00E-13	20573	22711	Y
				**G**C**LNIMGADL**	9/10 (90%)				
				**DLSVVGGTGDFFFSR**	14/15 (93%)				
13	**Disease resistance response/ dirigent protein**	Arabidopsis thaliana	At4g23690	**VIFDDPVTLDQNYLSKPV**S**R**	17/20 (85%)	1.00E-11	21412	24544	Y
				**SVVGGTGDFFMSR**	13/13 (100%)				
14	**Endochitinase class I, putative**	Arabidopsis thaliana	At2g43620	**RDTI**A**NVVTL**SV**F**NS**IFSK**	12/19 (63%)	2.00E-08	30378	25856	Y
				**QAFI**S**AAAQS**S**DAYK**	13/15 (87%)				
				**F**N**GLPLLTDPDMV**S**R**	13/15 (87%)				
15	**Endochitinase class I, putative**	Arabidopsis thaliana	At2g43620	**RDTI**A**NVVTL**SV**F**NS**IFSK**	12/19 (63%)	2.00E-08	30378	25969	Y
				**QAFI**S**AAAQS**S**DAYK**	13/15 (87%)				
				**F**N**GLPLLTDPDMV**S**R**	13/15 (87%)				
16	**Endochitinase class I, putative**	Arabidopsis thaliana	At2g43620	**RDTI**A**NVVTL**SV**F**NS**IFSK**	12/19 (63%)	7.00E-13	30378	25744	Y
				**QAFI**S**AAAQS**S**DAYK**	13/15 (87%)				
				**F**N**GLPLLTDPDMV**S**R**	13/15 (87%)				
				**PVLDQGFGATTR**	12/12 (100%)				
17	**Germin-like protein, subfamily 2**	Arabidopsis thaliana	At1 g02335	**YDPDALQDLCV**A**ADK**	14/15 (93%)	8.00E-07	23441	26543	Y
				**KIPGLNTLSV**	10/10 (100%)				
				**GEVFVFPR**	8/8 (100%)				
18	**Thaumatin-like protein/ osmotin**	Brassica rapa	AAN23104	**RAPNTLAEYALK**	12/12 (100%)	4.00E-04	incomplete	32060	
19	**Gamma-glutamyltranspeptidase 1**	Arabidopsis thaliana	At4g39640	**PPPAPANFIRPGK**	13/13 (100%)	4.00E-D5	61190	26,312*	Y
				**VPPPAPANFIRPGK**	14/14 (100%)				
				**QFIVQES**	7/7 (100%)				
20	**Endochitinase class I, putative**	Arabidopsis thaliana	At2g43620	**TI**A**NVVT**LSV**F**NS**IFSK**	10/17 (59%)	2.00E-20	30378	28613	Y
				**QAFI**S**AAAQS**S**DAYK**	13/15 (87%)				
				**FLGLPLLTDPD**F**VAR**	12/15 (80%)				
				**IQIT**SD**NYNYGAAGK**	12/17 (71%)				
21	**Xyloglucan:xyloglucosyl transferase**	Arabidopsis thaliana	At5g57560	**GQITN**DG**ELLTLSLDK**	14/16 (88%)	3.00E-36	32093	29928	Y
				**LVPGNSAGTVTTLYLK**	16/16 (100%)				
				**WFDPT**W**FHTYTIL**SD**NPQR**	16/19 (84%)				
				**IFTVDGTPIR**	10/10 (100%)				
				**NFES**V**GTLFPNNK**PFR	13/16 (81%)				
22	**Xyloglucan:xyloglucosyl transferase**	Arabidopsis thaliana	At5g57560	GQ**ITN**DG**ELLTLSLDK**	14/16 (88%)	3.00E-36	32093	30200	Y
				**LVPGNSAGTVTTLYLK**	16/16 (100%)				
				**WFDPT**VV**FHTYTIL**SD**NPQR**	16/19 (84%)				
				**IFTVDGTPIR**	10/10 (100%)				
				**NFES**V**GTLFPNNKPFR**	13/16 (81%)				
23	**Chitinase class IV, putative**	Arabidopsis thaliana	At2g43570	**PSFG**S**SISK**	8/9 (89%)	2.00E-05	29775	31326	Y
				**AAANSYPSFG**S**SISK**	14/15 (93%)				
				**L**N**ENLLASPEK**	10/11 (91%)				
				**VAQD**PPS**AFK**	7/10 (70%)				
24	**Peroxidase 25 precursor**	Arabidopsis thaliana	At2g41480	**STVESHFDSDPTISPGLLR**	19/19 (100%)	4.00E-09	35886	32665	Y
				**EASNLPSPLDSVAVQK**	16/16 (100%)				
				**VALDIGSPS**N**FD**V**SFFK**	15/17 (88%)				
25	**Legume lectin family protein**	Arabidopsis thaliana	At3g16530	**LA**V**EFDTFQNK**	10/11 (91%)	4.00E-06	30509	32360	Y
				**GYWVQTR**	7/7 (100%)				

Spot No	**Similarity to**	Organism	Acc. No.	**Sequence**	identity	E value	expected Mr	observed Mr	aS

26	**Legume lectin family protein**	Arabidopsis thaliana	At3g15356	**GDNL**F**FLGDAELG**	11/13(85%)	7.00E-15	29749	32819	Y
				**EFDTFKN**K	7/8 (88%)				
				**HVGININSMTS**N**VAEK**	15/16 (94%)				
				**TITIAPENVK**	10/10 (100%)				
27	**Chitinase class I**	Brassica napus	CAA43708	**DSFINAANTFPNFANSVTR**	19/19 (100%)	5.00E-11	28733	30200	Y
				**NGGNSGAVNAR**	11/11 (100%)				
28	**Legume lectin family protein**	Arabidopsis thaliana	At3g16530	**LA**V**EFDTFQNK**	9/10 (90%)	5.00E-06	30509	31763	Y
				**AGYWVQTR**	8/8 (100%)				
29	**Chitinase class I, putative**	Arabidopsis thaliana	At4g01700	**GFYPYEAFVEATR**	13/13 (100%)	1.00E-08	31464	32819	Y
				**GPIQLSWNYNYGQAGR**	16/16 (100%)				
30	**Peroxidase, putative**	Arabidopsis thaliana	At4g36430	**PGTVSCA**A**DLLTLAAR**	14/16 (88%)	4.00E-05	36164	34420	Y
				**Q**N**LSVLDI**V**SAA**K	10/13 (77%)				
				**GNISPLTGSSGEIR**	14/14 (100%)				
31	**Peroxidase, putative**	Arabidopsis thaliana	At4g36430	**DSSVLTGGP**C**WVVP**	13/14 (93%)	7.00E-18	36164	36508	Y
				**QGLDITDLVALSGSHTIGFSR**	21/21 (100%)				
				**NSDQVLFSS**	9/9 (100%)				
				**GNISPLTGSSGEIR**	14/14 (100%)				
32	**Peroxidase, putative**	Arabidopsis thaliana	At1g49570	**EAVVLTGGPF**MA**PVPLGR**	16/18 (89%)	9.00E-06	38030	41164	Y
				**GQPDPNLAASS**D**LLSK**	15/16 (94%)				
				**GNIGVFTGSDGVIR**	13/14 (93%)				
33	**Peroxidase ATP5a**	Arabidopsis thaliana	At1g49570	**EAVVLTGGPF**MA**PVPLGR**	16/18 (89%)	7.00E-05	38030	40065	Y
				**GNIGVFTGSDGVIR**	13/14 (93%)				
	**Peroxidase 59 precursor**	Arabidopsis thaliana	At5g19890	**LVEAYS**Q**SQSLFFR**	13/14 (93%)	6.00E-05	35023	40065	Y
34	**Serine carboxypeptidase S10 family protein**	Arabidopsis thaliana	At3g17180	**GFIVGNPLTDDEYDNK**	16/16 (100%)	1.00E-08	54145	40,498*	Y
35	**Curculin-like (mannose-binding) lectin**	Arabidopsis thaliana	At1g78830	**PLVYGG**DS**P**N**HDFR**	11/14 (79%)	2.00E-09	50343	39,640	Y
				**VVN**ND**EFG**D**YITEYDASYR**	16/19 (84%)				
				**GVTGFEILPNGNIVLHDK**	17/18 (94%)				
				**TG**Q**PLVYGG**DS**P**N**HDFR**	13/17 (76%)				
36	**Curculin-like (mannose-binding) lectin**	Arabidopsis thaliana	At1g78830	**PLVYGG**DS**P**N**HDFR**	11/14 (79%)	2.00E-09	50343	36,326*	Y
				**VVN**ND**EFG**D**YITEYDASYR**	16/19 (84%)				
				**GVTGFEILPNGNIVLHDK**	17/18 (94%)				
				**TG**Q**PLVYGG**DS**PNHDFR**	13/17 (76%)				
37	**Peroxidase, putative**	Arabidopsis thaliana	At4g33420	**PTL**S**AGLIR**	8/9 (89%)	1.00E-17	35975	37062	Y
				**NDFDNAYFNALQR**	13/13 (100%)				
				**SGVLFSDQTLFN**S**PFTR**	15/17 (88%)				
38	**Curculin-like (mannose-binding) lectin**	Arabidopsis thaliana	At1g78830	**PLVYGG**DS**P**N**HDFR**	11/14 (79%)	3.00E-08	50343	37,631*	Y
				**VVN**ND**EFG**D**YITEYDASYR**	16/19 (84%)				
				**ILPNGNIVLHDK**	12/1 (100%)				
				**TG**Q**PLVYGG**DS**P**N**HDFR**	13/17 (76%)				
39	**Curculin-like (mannose-binding) lectin**	Arabidopsis thaliana	At1g78830	**PLVYGG**DS**P**N**HDFR**	11/14 (79%)	3.00E-08	50343	35,967*	Y
				**VVN**ND**EFG**D**YITEYDASYR**	16/19 (84%)				
				**ILPNGNIVLHDK**	12/1 (100%)				
				**TG**Q**PLVYGG**DS**P**N**HDFR**	13/17 (76%)				
40	**Curculin-like (mannose-binding) lectin**	Arabidopsis thaliana	At1g78830	**PLVYGG**DS**P**N**HDFR**	11/14 (79%)	3.00E-08	50343	37,824*	Y
				**VVN**ND**EFG**D**YITEYDASYR**	16/19 (84%)				
				**ILPNGNIVLHDK**	12/1 (100%)				
				**TG**Q**PLVYGG**DS**P**N**HDFR**	13/17 (76%)				
41	**Curculin-like (mannose-binding) lectin**	Arabidopsis thaliana	At1g78830	**PLVYGG**DS**P**N**HDFR**	11/14 (79%)	3.00E-08	50343	36,326*	Y
				**VVN**ND**EFG**D**YITEYDASYR**	16/19 (84%)				
				**ILPNGNIVLHDK**	12/1 (100%)				
				**TG**Q**PLVYGG**DS**P**N**HDFR**	13/17 (76%)				
42	**Peroxidase 59 precursor**	Arabidopsis thaliana	At5g19890	**K**N**AIPNINSAR**	10/11 (91%)	0.003	35023	40280	Y
				**EAYSQSQSLFFR**	12/12 (100%)				
43	**Peroxidase, putative**	Arabidopsis thaliana	At4g33420	**PTLAAGLIR**	9/9 (100%)	1.00E-09	35975	37824	Y
				**NTAEKDSPANLSLR**	14/14 (100%)				
				**NDFDNAYFNALQR**	13/13 (100%)				
44	**Cyclase family protein**	Arabidopsis thaliana	At4g34180	**DYLSFAAF**D**ESPATHK**	15/16 (94%)	1.00E-28	28384	34586	Y
				**GLDYLSFAAF**D**ESPATHK**	17/18 (94%)				
				**EFDSSF**S**GFFTDGAK**	13/15 (87%)				
				**LIGLDYLSFAAF**D**ESPATHK**	19/20 (95%)				
				**GRDIIPVEALK**	11/11 (100%)				
45	**Peroxidase 21 (ATP2a)**	Arabidopsis thaliana	At2g37130	**DASLLLETAR**	10/10 (100%)	5.00E-18	36741	35614	Y
				**PTLDPDYALYLK**	11/12 (92%)				
				**ADNGYFHEQFSR**	12/12 (100%)				
				**LLSETNPLTGDQGEIR**	16/16 (100%)				
46	**Peroxidase, putative**	Arabidopsis thaliana	At4g33420	**PTLAAGLIR**	9/9 (100%)	8.00E-40	35975	38018	Y
				**GFTPQDVVALSGAHTLGVAR**	20/20 (100%)				
				**LTTPDSSFDS**S**F**V**NTLTK**	13/18 (72%)				
				**FDNAYFNALQ**R	10/11 (91%)				
				**SGVLFSDQTLFNTPATR**	17/17 (100%)				
47	**Peroxidase, putative**	Arabidopsis thaliana	At4g33420	**DNTAEKDSPANLSLR**	15/15 (100%)	1.00E-25	35975	37062	Y
				**GFTPQDVVALSGAHTLGVAR**	20/20 (100%)				
				**NDFDNAYFNALQ**R	12/13 (92%)				
				**LFSDQTLFNTP**	11/11 (100%)				
48	**Peroxidase 21 (ATP2a)**	Arabidopsis thaliana	At2g37130	**DASLLLETAR**	10/10 (100%)	8.00E-09	36741	36691	Y
				**LLSETNPLTGDQGEIR**	16/16 (100%)				
49	**Beta-1,3-glucanase (BG3)**	Arabidopsis thaliana	At3g57240	**L**L**LDVPNPDLQR**	11/12 (92%)	3.00E-1B	30712	36691	N
				**YISVGNEVQPS**	11/11 (100%)				
				**FVLPAMQNI**D**R**	10/11 (91%)				
				**TYVNNLIQTVK**	11/11 (100%)				

Spot No	**Similarity to**	Organism	Acc. No.	**Sequence**	identity	E value	expected Mr	observed Mr	aS

50	**Beta-1,3-glucanase (BG3)**	Arabidopsis thaliana	At3g57240	**L**L**LDVPNPDLQR**	11/12(92%)	3.00E-18	30712	37250	N
				**YISVGNEVQPS**	11/11 (100%)				
				**FVLPAMQNI**D**R**	10/11 (91%)				
				**TYVNNLIQTVK**	11/11 (100%)				
51	**Polygalacturonase inhibitor protein 2**	Brassica napus	AAM94869	**NFLQFDLSR**	9/9 (100%)	5.00E-05	37117	40280	Y
				**LQEFDTYSYFHNK**	13/13 (100%)				
52	**Peroxidase 59 precursor**	Arabidopsis thaliana	At5g19890	**P**Y**LVQIVR**	7/8 (88%)	2.00E-06	35023	41850	Y
				**GFEV**V**DTIK**	8/9 (89%)				
				**LVEAYS**Q**SQSLFFR**	13/14 (93%)				
53	**Peroxidase 59 precursor**	Arabidopsis thaliana	At5g19890	**P**Y**LVQIVR**	7/8 (88%)	5.00E-04	35023	43793	Y
				**LVEAYS**Q**SQSLFFR**	13/14 (93%)				
54	**Peroxidase 59 precursor**	Arabidopsis thaliana	At5g19890	**P**Y**LVQIVR**	7/8 (88%)	2.00E-06	35023	43793	Y
				**GFEV**V**DTIK**	8/9 (89%)				
				**LVEAYS**Q**SQSLFFR**	13/14 (93%)				
55	**Subtilase family protein**	Arabidopsis thaliana	At1g20160	**AVASAYG**T**FPTTVIDSK**	16/17 (94%)	1.00E-17	81480	56,025*	Y
				**SILKPDITAPGV**A**ILAAWTG**	19/20 (95%)				
				**LTYQV**T**VSATA**	10/11 (91%)				
56	**Subtilase family protein**	Arabidopsis thaliana	At1g20160	**SPVYPLIHGK**	10/10 (100%)	2.00E-05	81480	56,025*	Y
				**ASAYG**T**FPTTVIDSK**	14/15 (93%)				
				**VSVETP**AYQ**IQVTPEK**	13/16 (63%)				
				**VFGALT**	6/6 (100%)				
57	**Curculin-like (mannose-binding) lectin**	Arabidopsis thaliana	At1g78850	**KVPVD**E**QF**L**VVN**K	10/13 (77%)	8.00E-16	49051	50573	Y
				**TDYSPIEYNPDVR**	13/13 (100%)				
				**ILENGN**F**VIYDSS**V**K**	13/15 (87%)				
				**SVNT**D**GPYSLV**F**E**D**K**	12/15 (80%)				
				**TPKPIA**VR**E**G**YEFFSK**	12/16 (75%)				
58	**Polygalacturonase/ glycoside hydrolase 28**	Arabidopsis thaliana	At3g61490	**DATLLAAQDL**E**EYP**V**LK**	14/17 (82%)	1.00E-10	51939	56471	Y
				**DEYGI**A**FG**F**PTK**	10/12 (83%)				
				**IALGSE**F**SGGIEDVR**	14/15 (93%)				
59	**Subtilase family protein**	Arabidopsis thaliana	At1g20160	**SPVYPLIHGK**	10/10 (100%)	1.00E-10	81480	58,848*	Y
				**ASAYG**T**FPTTVIDSK**	14/15 (93%)				
				**S**L**HPTW**C**PSAIR**	10/12 (83%)				
				**VSVETP**AYQ**IQVTPEK**	13/16(81%)				
				**LTYQV**T**VSA**ADD**DV**	10/14(71%)				
				**VFGALT**	6/6 (100%)				
60	**Subtilase family protein**	Arabidopsis thaliana	At1g20160	**SPVYPLIHGK**	10/10 (100%)	1.00E-10	81480	56,025*	Y
				**ASAYG**T**FPTTVIDSK**	14/15 (93%)				
				**S**L**HPTW**C**PSAIR**	10/12 (83%)				
				**VSVETP**AYQ**IQVTPEK**	13/16(81%)				
				**LTYQV**T**VSA**ADD**DV**	10/14(71%)				
				**VFGALT**	6/6 (100%)				
61	**Curculin-like (mannose-binding) lectin**	Arabidopsis thaliana	At1g78850	**KVPVD**E**QF**L**VVN**K	10/13 (77%)	6.00E-11	49051	50573	Y
				**TDYSPIEYNPDVR**	13/13 (100%)				
				**ILENGN**F**VIYDSS**V**K**	13/15 (87%)				
				**SVNT**D**GPYSLV**F**E**D**K**	12/15 (80%)				
				**TPKPIA**VR**E**G**YEFF**S**K**	12/16 (75%)				
62	**Curculin-like (mannose-binding) lectin**	Arabidopsis thaliana	At1g78850	**KVPVD**E**QF**L**VVN**K	10/13 (77%)	6.00E-11	49051	51999	Y
				**TDYSPIEYNPDVR**	13/13 (100%)				
				**ILENGN**F**VIYDSS**V**K**	13/15 (87%)				
				**SVNT**D**GPYSLV**F**E**D**K**	12/15 (80%)				
				**TPKPIA**VR**E**G**YEFF**S**K**	12/16 (75%)				
63	**Subtilase family protein**	Arabidopsis thaliana	At1g20160	**AVASAYG**T**FPTTVIDSK**	16/17 (94%)	3.00E-07	81480	56,926*	Y
				**TVTNVG**G**D**R**AVY**K	10/13 (77%)				
				**VFGALT**	6/6 (100%)				
64	**Polygalacturonase/ glycoside hydrolase 28**	Arabidopsis thaliana	At3g61490	**DEYGI**A**FG**F**PTK**	10/12 (83%)	9.00E-08	51939	5786B	Y
				**IALGSE**F**SGGIEDVR**	14/15 (93%)				
				**WTLLAAQDL**E**EYP**V**LK**	14/16 (88%)				
65	**Subtilase family protein**	Arabidopsis thaliana	At1g20160	**ESDVVLGGSK**	9/10 (90%)	5.00E-08	81480	81188	Y
				**SPVYPLIHGK**	10/10 (100%)				
				**AVASAYG**T**FPTTVIDSK**	16/17 (94%)				
				**S**L**HPTW**C**PSAIR**	10/12 (83%)				
				**TVTNVG**G**D**R**AVY**K	10/13 (77%)				
				**LTYQV**T**VSA**	8/9 (89%)				
66	**Subtilase family protein**	Arabidopsis thaliana	At1g20160	**ESDVVLGG**S**K**	9/10 (90%)	5.00E-08	81480	79849	Y
				**SPVYPLIHGK**	10/10 (100%)				
				**AVASAYG**T**FPTTVIDSK**	16/17 (94%)				
				**S**L**HPTW**C**PSAIR**	10/12 (83%)				
				**TVTNVG**G**D**R**AVY**K	10/13 (77%)				
				**LTYQV**T**VSA**	8/9 (89%)				
67	**Subtilase family protein**	Arabidopsis thaliana	At1g20160	**ESDVVLGG**S**K**	9/10 (90%)	6.00E-07	81480	81188	Y
				**AVASAYG**T**FPTTVIDSK**	16/17 (94%)				
				**TVTNVG**G**D**R**AVY**K	10/13 (77%)				
				**LTYQV**T**VSA**	8/9 (89%)				
				**VFGALT**	6/6 (100%)				
68	**Cucumisin-like serine protease (ARA12)**	Arabidopsis thaliana	At5g67360	**VEGASLLG**F**ASGTAR**	14/15 (93%)	1.00E-06	79415	78587	Y
				**DGVAIGAFAA**F**ER**	12/13 (92%)				
				**PALAILGNGK**	10/10 (100%)				
				**LNYPSFAVNVDG**S**GAYK**	16/17 (94%)				
				**TVTFTVDSSK**	10/10 (100%)				
69	**Germin-like protein**	Arabidopsis thaliana	At3g05950	**FTSGLNIAGNTIN**	13/13 (100%)	7.00E-05	24736	76,260**	Y
				**AFQLDVNVV**R	9/10 (90%)				
